# CAP/ACMG proficiency testing for biochemical genetics laboratories: a summary of performance

**DOI:** 10.1038/gim.2017.61

**Published:** 2017-06-29

**Authors:** the College of American Pathologists and American College of Medical Genetics and Genomics, Biochemical and Molecular Genetics Resource Committee, Devin Oglesbee, Tina M Cowan, Marzia Pasquali, Timothy C Wood, Karen E Weck, Thomas Long, Glenn E Palomaki

**Affiliations:** 1 Department of Laboratory Medicine and Pathology, Mayo Clinic College of Medicine, Rochester, Minnesota, USA; 2 Department of Pathology, Stanford University, School of Medicine, Palo Alto, California, USA; 3 Department of Pathology, University of Utah School of Medicine, Salt Lake City, Utah, USA; 4 Greenwood Genetics Center, Greenwood, South Carolina, USA; 5 Department of Pathology and Laboratory Medicine, University of North Carolina at Chapel Hill, Chapel Hill, North Carolina, USA; 6 Department of Biostatistics, College of American Pathologists, Northfield, Illinois, USA; 7 Department of Pathology and Laboratory Medicine, Women and Infants Hospital, Alpert Medical School of Brown University, Providence, Rhode Island, USA

**Keywords:** analytic sensitivity, biochemical genetics, clinical sensitivity, inborn errors of metabolism; proficiency testing

## Abstract

**Purpose:**

Testing for inborn errors of metabolism is performed by clinical laboratories worldwide, each utilizing laboratory-developed procedures. We sought to summarize performance in the College of American Pathologists’ (CAP) proficiency testing (PT) program and identify opportunities for improving laboratory quality. When evaluating PT data, we focused on a subset of laboratories that have participated in at least one survey since 2010.

**Methods:**

An analysis of laboratory performance (2004 to 2014) on the Biochemical Genetics PT Surveys, a program administered by CAP and the American College of Medical Genetics and Genomics. Analytical and interpretive performance was evaluated for four tests: amino acids, organic acids, acylcarnitines, and mucopolysaccharides.

**Results:**

Since 2010, 150 laboratories have participated in at least one of four PT surveys. Analytic sensitivities ranged from 88.2 to 93.4%, while clinical sensitivities ranged from 82.4 to 91.0%. Performance was higher for US participants and for more recent challenges. Performance was lower for challenges with subtle findings or complex analytical patterns.

**Conclusion:**

US clinical biochemical genetics laboratory proficiency is satisfactory, with a minority of laboratories accounting for the majority of errors. Our findings underscore the complex nature of clinical biochemical genetics testing and highlight the necessity of continuous quality management.

## Introduction

Clinical biochemical genetics is a medical specialty devoted to diagnosing and treating inborn errors of metabolism (IEMs). IEMs encompass a heterogeneous group of genetic conditions including disorders of amino acid (AA; e.g., phenylketonuria), organic acid (OA; e.g., methylmalonic aciduria, MMA), mucopolysaccharide (MPS; e.g., Hurler syndrome), and fatty acid metabolism (e.g., medium-chain acyl-CoA dehydrogenase, MCAD, deficiency). Although individually rare, their estimated combined incidence is as high as 1:800–1:2,500 births.^1, 2^ Symptoms often appear early in life, but clinical outcomes are optimized by an early diagnosis and therapy instigation before irreversible damage has occurred.^3^

An IEM diagnosis is established through high-complexity, laboratory-developed tests performed by a relatively small number of laboratories worldwide, called clinical biochemical genetics laboratories (BGLs). Methods vary between laboratories, but they typically include high-pressure liquid chromatography, gas chromatography/mass spectrometry and/or liquid chromatography–tandem mass spectrometry (LC-MS/MS). There are no US Food and Drug Administration–cleared tests for biochemical genetics testing, a commonality shared with molecular genetics laboratories and DNA-based testing for rare heritable conditions. Despite the nonstandardized nature of laboratory testing, molecular genetic testing quality is very high.^4, 5, 6, 7, 8, 9, 10, 11^ Nevertheless, biochemical genetic testing poses unique challenges to the clinical laboratory, including the innate task of assessing multiple analytes in a single test, and the inter- and intrapatient metabolic variability due to genotype, clinical status, or other intrinsic and extrinsic factors. Thus, it is vital that laboratories perform extensive validation and ongoing test monitoring to ensure the highest quality for patient care.

Participation in proficiency testing (PT) is associated with improved laboratory quality, particularly in the laboratory-developed test setting.^12, 13, 14^ External PT schemes for BGL testing were first introduced in the United States in the 1980s, with a review of outcomes highlighting the importance of ongoing training, education, and quality oversight in the area of IEM diagnosis.^15, 16^ Comprehensive PT is also offered by the European Research Network for Evaluation and Improvement of Screening, Diagnosis and Treatment of Inherited Disorders of Metabolism (ERNDIM), which reported a positive correlation between participation and laboratory quality.^17^ In 1993, the College of American Pathologists (CAP), in collaboration with the American College of American Genetics and Genomics (ACMG), introduced a BGL PT program that included the analysis of AAs, OAs, and MPS. The program was expanded in 2004 to include plasma acylcarnitine (AC) performance.

Here, we present an analysis of the CAP/ACMG Biochemical Genetics Resource Committee PT program from more than 10 years of surveys (2004 through 2014). The analysis was restricted to laboratories that had participated in at least one PT survey since 2010. This was done to allow for estimates of recent laboratory performance, but it also permitted the examination of earlier PT performance in a subset of active laboratories.

## Materials and methods

### BGL proficiency testing survey

The PT survey consists of five specimens distributed twice yearly (an A and B distribution) to laboratories in the United States and abroad. Each mailing contained one specimen for every analysis, including plasma or urine AA, urine OA, plasma AC, and urine MPS. A fifth specimen, excluded from this analysis, is an ungraded educational challenge focusing on less frequently encountered areas of AA, OA, or AC testing. This report includes results from 21 BGL surveys from the 2004-B through 2014-B mailings.

Most specimens were authentic plasma or urine samples from patients with clinically confirmed diagnoses. Occasionally, samples were from unaffected individuals, pooled samples, or spiked with one or more compounds to mimic a metabolic disorder. All plasma samples used had negative test results for hepatitis B surface antigen, hepatitis C antibody, and HIV antigen/antibody. Challenges were pretested by multiple reference laboratories to verify suitability. Specimens were stored at −20 °C or lower, and aliquots were shipped on dry ice. A clinical history accompanied each challenge. Participants reported all diagnostically relevant analyte(s), the most likely diagnosis (clinical interpretation) based on analytic results and clinical history, and by selecting answers from a predefined list. Information about testing methodology and results of analyte quantitation were requested but not required or graded. Laboratories returned results within 30 days via an online result submission form or facsimile.

### Data collection and analysis

Data were extracted from the CAP data management system and de-identified. These data encompassed participant results, including those returned after the required submission date and excluded from original Participant Summary Reports. Each challenge’s analytic and interpretative results were graded as “Acceptable” or “Unacceptable” based on participant consensus (>80%) or, in a few instances, on the consensus of reference laboratory results. Missing analytic results or missing clinical interpretations were not graded and were excluded from analysis. Members of the CAP/ACMG Biochemical and Molecular Genetics Resource Committee reviewed results and approved grading criteria. Acceptable results were based on either the identification of a well-recognized, analytical profile, or a pathognomonic analyte level, depending on the circulated specimen, and correlation with the provided clinical scenario. A two-dimensional matrix of the graded responses stratified by time (columns) and participants (rows) was created. These “heat maps” were used to compute analytic sensitivity (the proportion of abnormal analytes correctly identified) and clinical sensitivity (the proportion of disorders correctly diagnosed) for all challenges.

Calculations of clinical sensitivity included separate analyses for two types of false-negative results. One type occurs when participants recognize a specimen as having an abnormal clinical interpretation but report the incorrect disorder (i.e., not the intended response). The second type occurs when participants fail to recognize any clinical disorder and interpret the result as “Normal, unaffected.” This latter type is the classic false negative. The MPS survey had multiple challenges from unaffected individuals (six in total) and these were used to calculate analytic specificity (the proportion of normal specimens with no abnormal analytes reported) and clinical specificity (the proportion of normal specimens correctly diagnosed as normal). Rates were compared using the chi-squared test with a two-tailed significance level of 0.05. The 95% confidence intervals for proportions were computed using the adjusted Wald asymptotic method.

## Results

### Participants and tests challenged

A total of 150 laboratories participated in the program for at least one of the AA, OA, AC, and MPS challenges since 2010, including 97 US and 53 international laboratories (North America, South America, Asia, and Europe). Of these, 35 (23%) reported results for all four schemes (AA, OA, AC, and MPS), while 49 (33%) participants reported results for only three schemes (25 for AA, OA, and AC and 23 for AA, OA, and MPS). Among the 38 (25%) participants reporting results for two schemes, the most common pair was AA and OA (26 participants) and among the 28 (19%) reporting results for only one scheme, the most common was AA (22).

### AA proficiency testing

The AA challenges included specimens from individuals with phenylketonuria, maple syrup urine disease, various urea cycle defects, cystinuria, and other AA transport disorders (Supplementary Table S1 online). Results from 2013-A were excluded due to sample degradation likely related to storage. A total of 141 laboratories (91 US, 50 international) provided responses, with the number of participants remaining essentially constant since 2010 at around 95 participants. Most participants (75% in 2010) used AA analyzers, but this proportion dropped to 68% in 2014. LC-MS/MS was reported by three laboratories in 2010 and by nine laboratories in 2014.

**Figure 1:**
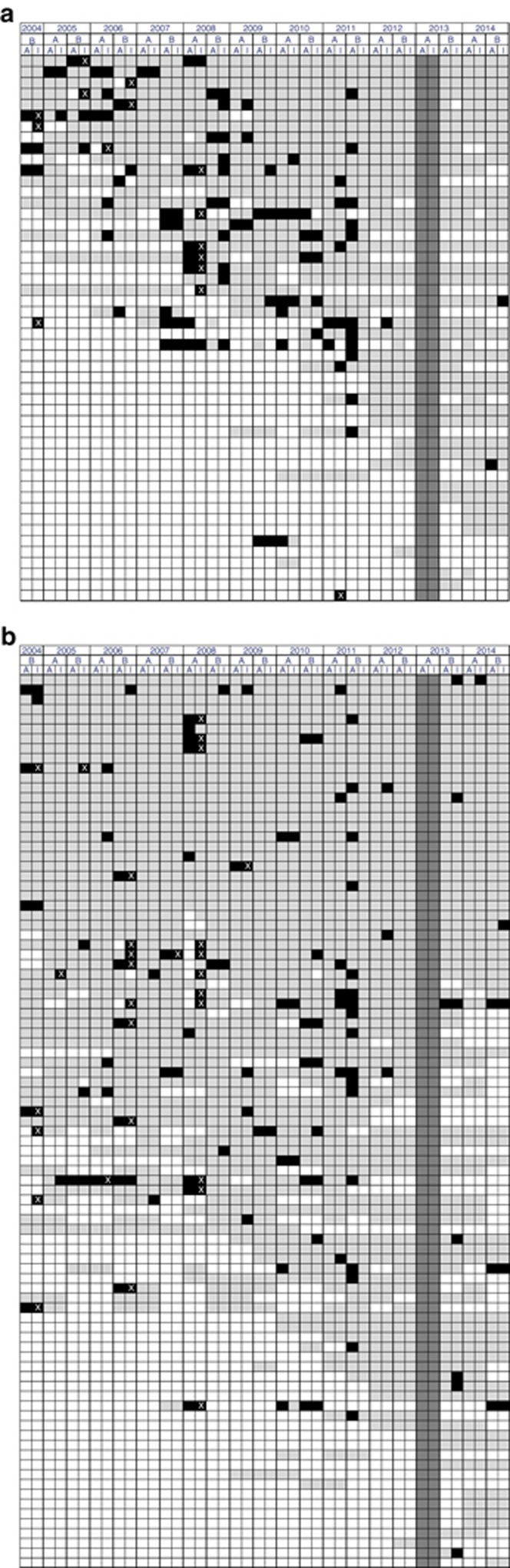
**Heat maps of CAP proficiency testing results for amino acid challenges from 2004-B through 2014-B.** (**a**) Heat map for amino acid proficiency testing for 50 international participants. (**b**) Heat map for amino acid proficiency testing for 109 US participants. Each row represents results from a single laboratory while each column represents a specific response. Each pair of columns depicts grading results for the analytic (A) and interpretive (I) component of the challenge (e.g., columns 1 and 2 show analytic and interpretive performance for the B distribution in 2004). Gray boxes indicate correct responses; black boxes, incorrect responses; white boxes, no response (or no participation). For clinical interpretations (I), a white X indicates that the incorrect interpretation was “Normal, unaffected” (i.e., false-negative result); while a solid black box indicates that an abnormality was recognized but was incorrect. The two solid gray columns indicate an ungraded sample (see text).

AA PT results are summarized in the heat map (Figure 1), which includes participant-specific performance over time for both analytic and clinical interpretations (see also Supplementary Figure S1). The overall analytic sensitivity for AA testing was 93.4% (1,552/1,661 responses), with the highest sensitivities (100%) observed for conditions related to phenylalanine, citrulline, and arginine metabolism, and the lowest for abnormalities in β-alanine (87.3%) and α-aminoadipic acid (86.2%). Stratified by geographic region, the analytic sensitivity was higher (*P*<0.001) among US (95.1%, 1,122/1,180) than among international participants (89.4%, 430/481). Among US participants, 53 (58%) had no incorrect responses for analytic sensitivity. Four of these 91 laboratories (4%) accounted for 14 errors (between 3 and 6 each), representing 24% of all analytical errors. Among international participants, 23 (46%) had no incorrect responses for analytic sensitivity; six of these 50 laboratories (12%) accounted for 22 errors (between 3 and 5 each) representing for 43% of all analytic errors.

The overall clinical sensitivity for all participants was 91.0% (1,514/1,663), but the clinical sensitivity was significantly higher among US participants (92.9% 1,103/1,188) than international participants (86.5% 411/475) (*P*<0.001). Clinical sensitivity was highest for more easily recognizable conditions (phenylketonuria, 100%) and lowest for combined homocystinuria/methylmalonic aciduria (2008A, 76.3%). Despite compelling clinical scenarios, clinical sensitivity was also lower for several challenges where the differential diagnosis was broad, including 83.6% for homocystinuria (2006A, cystathionine-β-synthase deficiency) and 84.0% for primary lactic acidemia (2008B). Incorrect clinical interpretations were stratified by incorrect diagnosis (e.g., a sample with elevated citrulline mistakenly diagnosed as ornithine transcarbamylase deficiency rather than citrullinemia) versus “normal” clinical interpretation. This latter group of false negatives represented 2.6% (44/1,663) of all interpretations over the 10-year period. Among US laboratories, the rate of 2.4% (29/1,188) was not different from the 3.2% (15/475) found among international laboratories (*P*=0.41). Since 2010, however, laboratories have improved with only one false-negative result occurring in more than 850 distinct clinical interpretations. A summary of AA performance results along with 95% confidence intervals is provided in Table 1.

**Figure 2:**
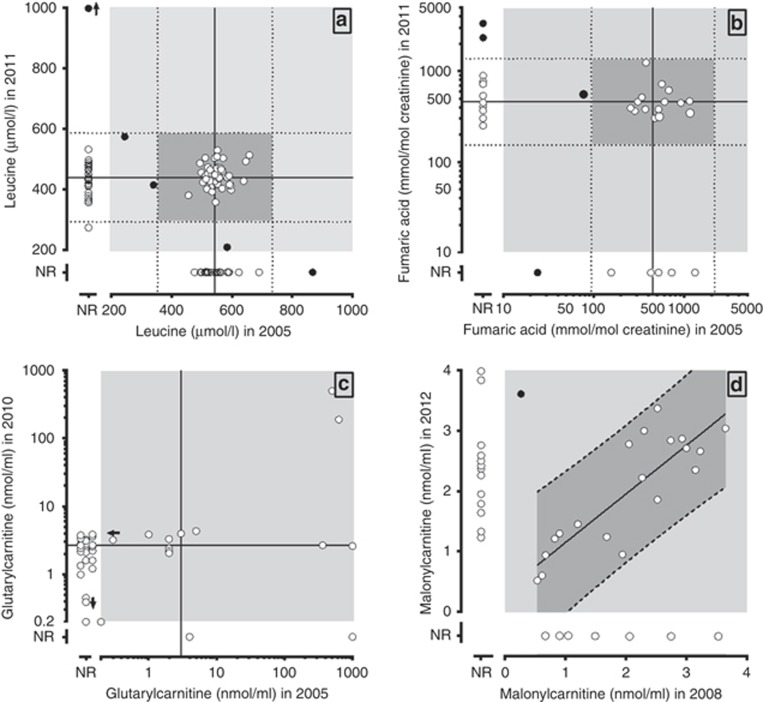
**Comparisons of reported analyte quantitation from identical sample challenges provided in different years.** (**a**) The reported leucine quantitation from the same maple syrup urine disease sample tested in 2005 (*n*=93) and 2011 (*n*=95). Lines represent the median (solid) and ±3 s.d. (dotted) values of participant responses for each year, with the darker gray box indicating acceptable performance. Sixty participants responded to both surveys. Open circles indicate the results falling within the computed prediction limits while dark circles are those results outside those limits for one or both distributions. Open circles just to the left of the *y*-axis or just above the *x*-axis represent results from laboratories reporting only in 2005 or 2011, respectively. An arrow next to an observation indicates the reported value is higher (or lower) than the range depicted. (**b**) Fumarate quantitation from the same fumarase deficiency sample tested in 2005 (25 responses) and in 2011 (29 responses); 16 participants responded to both surveys. Lines, symbols, and shading are the same as for (**a**). (**c**) Glutarylcarnitine quantitation from the same specimen submitted in both 2008 (15 responses) and 2012 (35 responses). There were insufficient data to compute prediction limits for 2008. Lines, symbols, and shading are the same as for (**a**). An arrow next to an observation indicates the reported value is higher (or lower) than the range depicted. (**d**). Malonylcarnitine quantitation from the same specimen submitted in both 2008 (26 responses) and 2012 (33 responses). Although individual laboratories tended to provide similar results for both challenges, those results differed significantly between laboratories. Thus, the prediction limits fall around a line rather than a point. Lines, symbols, and shading are the same as for (**a**).

Two challenges with a maple syrup urine disease specimen were distributed in 2005 and 2011. Leucine quantitation (Figure 2a) was 18% lower in the later distribution (443 vs. 541 μmol/l) but the coefficients of variation were comparable (5.4 vs. 8.9%). Despite the leucine concentration reductions, results were still significantly elevated and consistent with a diagnosis of maple syrup urine disease. Among the 60 laboratories participating in both challenges, 57 (95%) reported results within the 95% prediction interval (mean±1.96 standard deviations).

### OA proficiency testing

OA challenges included conditions such as MMA and propionic acidemia along with others (Supplementary Table S1). A total of 113 laboratories (73 US, 40 international) provided responses, with the participation increasing from 75 in 2010 (53 US, 22 international) to 85 in 2014 (57 US, 28 international). Analytic methods did not change substantially over the 10-year study period. In 2014, 98% of participants used gas chromatography/mass spectrometry, with 94% employing solvent extraction for sample preparation (50% ethylacetate, 40% ethylacetate/ether, 4% acetonitrile or other solvents) and 6% using solid-phase extraction. Single laboratories reported using gas chromatography/mass spectrometry or LC-MS/MS methods.

OA performance results are summarized in Tables 1 and 2, and in a heat map (Supplementary Figure S2). The overall analytic sensitivity was 92.7% (1,311/1,415), with a higher rate in US (93.5% 942/1,007) versus international (90.4% 369/408) laboratories (*P*=0.043). Analytic performance was excellent for challenges with prominent abnormalities, such as MMA (2012B, 100%), 5-oxoprolinuria (2009A, 100%), and fumarase deficiency (2005A, 96.6% and 2011A, 95.0%). Performance was poor for specimens with modest metabolite elevations, such as MCAD deficiency (2010A, 83.6% and 2013A, 81.5%). However, the circulation of specimens originating from different donors with divergent clinical statuses complicated the investigation of poor performance, as was the case for MCAD deficiency.

The overall clinical sensitivity was 90.5% (1,301/1,437), and was significantly higher among US (92.9% 947/1,019) than international (84.7% 354/418) participants (*P*<0.001). Interpretive performance was excellent for challenges that included specimens with prominent metabolite elevations and poor for challenges containing specimens with complex or subtle findings such as glutaric acidemia type II (2008B, 85.3%) or isobutyryl-CoA dehydrogenase deficiency (2006B, 75.0%). Among 73 US participants, 36 (49%) had no interpretive errors; 29 (40%) had one or two errors, and one laboratory accounted for nine errors. From 72 total errors, 43 (60%) reflected an incorrect diagnosis and 29 (40%) were false negatives (normal diagnosis). Among 40 international participants, 12 (30%) had no incorrect clinical interpretations, but two had four errors and one had five errors. In this group, there were 64 errors, of which 35 (55%) were incorrect diagnoses but 29 (45%) were identified as normal.

A single OA specimen of fumarase deficiency was distributed in 2005 and 2011, with quantitative data reported by 25 and 30 participants, respectively (Figure 2b). Median fumaric acid concentrations were 451 and 457 mmol/mol creatinine for the 2005 and 2011 mailings, respectively. The results indicate satisfactory agreement between participants reporting quantitative values. There were a handful of outliers, yet their results were still indicative of fumarase deficiency.

### AC proficiency testing

AC challenges included disorders of fatty acid oxidation such as MCAD deficiency, very long-chain acyl-CoA dehydrogenase deficiency, and long-chain hydroxyacyl-CoA dehydrogenase deficiency, as well as primary carnitine transporter deficiency, and organic acidemias such as MMA and propionic acidemia (Supplementary Table S1). A total of 65 laboratories (42 US, 23 international) provided results, with the number of participants rising from 43 in 2010 to 52 in 2014. By 2014, all laboratories were using (LC-MS/MS) with 91% utilizing butyl esters and the remainder using methyl esters or underivatized compounds.

AC performance results are summarized in Tables 1 and 2 and in a heat map (Supplementary Figure S3). The overall analytic sensitivity was 91.8% (663/722), with 93.1% (471/506) in US laboratories and 88.9% (192/216) in international laboratories (*P*=0.083). Analytic sensitivity was 100% for several challenges including MCAD, MMA/propionic acidemia, and a recent very long-chain acyl-CoA dehydrogenase deficiency challenge (2014A). The lowest analytic sensitivity (64.5%) was seen for the 2009A challenge of long-chain hydroxyacyl-CoA dehydrogenase deficiency. Among all 65 participants, 29 (45%) had no analytical errors. One or two errors were made by 19 (29%) and 12 (18%) participants, respectively. Five laboratories (three US and two international, or 8%) reported three or more errors, and these participants accounted for 16 of the 59 total analytic errors (27%).

The overall clinical sensitivity for AC PT was 88.0% (650/739), but this rate was higher among US laboratories (90.1% 462/513) compared to international laboratories (83.2% 188/226) (*P*=0.010). For three challenges (2005B, 2006A, and 2014A), all laboratories identified the correct abnormal analyte(s) (100% analytic sensitivity) and also provided the correct clinical interpretation (100% clinical sensitivity). Challenges with lower clinical sensitivities either had nonspecific analytic findings compatible with more than one disorder (e.g., elevated C5OH in β-ketothiolase deficiency (77.5%); low free carnitine in carnitine uptake defect (75.7%)), or had only modest analyte elevations, including both challenges containing specimens from known patients with long-chain hydroxyacyl-CoA dehydrogenase deficiency (75.0% and 74.4%). Among the 65 participants, 23 (35%) had no interpretive errors during the 10-year period and 31 (48%) had one or two errors. Eleven laboratories (17%) had three or more errors; one with five and two with six. Among these 89 errors, a subset reported that there was no disorder identified; a false-negative result. The rates for this type of error differed significantly by location (*P*=0.001), with a 2.7% (14/513) and 8.0% (18/226) difference in the US and international participants, respectively (Table 2).

Among challenges constituting a specimen submitted for PT more than once, and for which results included quantitative AC values, concentrations for significant AC results are shown in Figure 2c. Broad interlaboratory variation was observed for glutarylcarnitine (C5DC) in 2005 (four results >300 nmol/ml and 10 between 1 and 5 nmol/ml), with significant improvement in 2010 (one result of 189 nmol/ml and 32 between 1 and 5 nmol/ml). Interlaboratory variability was also observed for malonylcarnitine (C3DC) measurements obtained from a specimen submitted for challenges in 2008 and 2012 (Figure 2d). While there was high interlaboratory variability in both 2008 and 2012, one outlier was identified among the 19 laboratories participating in both challenges. The remaining results were highly correlated (*N*=18, *r*=0.84), indicating low intralaboratory variability and the need for further standardization efforts.

### MPS proficiency testing

MPS challenges included Hurler/Hunter (MPS-I/II, graded together), Sanfilippo (MPS-III), Morquio (MPS-IV), and Sly (MPS-VII) syndromes, as well as six normal individuals (Supplementary Table S1). Results were reported for a screening assay for total MPS and/or a fractionation assay (elevations of keratan sulfate, dermatan sulfate, heparan sulfate, and chondroitin sulfate). Overall, 72 laboratories (42 US and 30 international) participated in the PT. Dimethylmethylene blue binding assay (50%) was the most common screening method, followed by toluidine blue (16%), Berry spot test (11%), and Alcian blue spot test (5%). Thin-layer chromatography and electrophoresis were reported among fractionation methods in similar frequencies, but LC-MS/MS increased to 11% by 2014.

MPS performance is summarized in Tables 1 and 2 and in a heat map (Supplementary Figure S1). Two MPS-VII samples (2008A, 2011A) were excluded from analysis due to the lack of participant consensus (<80%). The combined analytic sensitivity for all screening methods was 91.2% (497/545) with similar (*P*=0.060) performance by US (93.0%, 318/342) and international participants (88.2%, 179/203). Screening performance was best for MPS-I/II (analytic sensitivity 96.5% 164/170), but poorer for MPS-III (88.1% 185/210). Analytic sensitivity for the fractionation assays was 87.2% (307/352) and performance was significantly higher (*P*=0.001) for US (91.4%, 213/233) than for international (79.0%, 94/119) participants. The sensitivity of fractionation assays was highest for MPS-III (95.7% 132/138) and lowest for MPS-IV (78.8% 63/80). Screening assays were more sensitive than fractionation methods for detecting an MPS abnormality. In particular, discrepancies were noted between the screening and fractionation methods for the 2010A challenge of MPS-II (100 vs. 83%) and the 2010B challenge of MPS-IV (98 vs. 73%)

The overall clinical sensitivity was 82.4% (333/404), with a significant difference (*P*<0.001) between US (88.3%, 227/257) and international (72.1%, 106/147) laboratories. Clinical sensitivity was highest among MPS-III (Sanfilippo syndrome) challenges (88.2% 135/153). The MPS-VII (Sly syndrome) challenges were the most problematic; two PT challenges lacked consensus for grading and the third had an overall clinical sensitivity of 79.3% (23/29).

For screening assays, 86% of laboratories (62/72) made one or fewer analytical errors (56% had no errors, 31% had one error). In addition, 10 laboratories (14%) made two or more errors apiece and were responsible for 26 of the 48 errors (54%). For the 49 laboratories utilizing fractionation assays, 33% (16) had no errors and 51% (25) had one error. The remaining eight laboratories (16%) made two or more errors apiece and were responsible for 20 of 45 errors (44%). Of the 56 laboratories reporting interpretations, 70% (39/56) had none or one interpretative error. The remaining 17 (30%) laboratories with two or more errors were responsible for 70% (50 /71) of the errors.

Analytic specificity, calculated from results of the six normal samples, was 99.6% (254/255) for the screening assay and 93.9% (139/148) for the fractionation assay. Stratifying fractionation results by laboratory location revealed an analytic specificity of 96.1% (99/103) among US and 88.9% (40/45) among international participants (*P*=0.091). Overall, 85% (23/27) of US and 79% (15/19) of international laboratories had no analytic specificity errors. The corresponding clinical specificity was 89.0% (169/190) with no significant difference by laboratory location.

## Discussion

The vast majority of PT participants accurately identified diagnostic abnormalities and correctly interpreted corresponding genetic conditions. Performance was excellent for challenges with prominent and characteristic biochemical abnormalities, including samples from patients with classic genetic conditions such as phenylketonuria, MMA, and very long-chain acyl-CoA dehydrogenase deficiency. In contrast, performance waned when challenges involved more subtle analytical findings (e.g., minimally elevated C16-OH AC concentrations seen in long-chain hydroxyacyl-CoA dehydrogenase deficiency), diagnoses relying on the recognition of a complex, multianalyte pattern (e.g., multiple acyl-Co dehydrogenase deficiency) and ultrarare conditions (e.g., mevalonic aciduria). Importantly, the proportion of participants completely missing a definitive diagnosis was low, and the majority of conditions were recognized as having abnormal biochemical profiles, even if the precise diagnosis provided was deemed unacceptable in the PT scheme.

Among the selected laboratories, performance over this study period was steadily higher among US laboratories than international laboratories. The reason for this tendency is unknown. Several factors between laboratories may account for this correlation. In particular, there exist specialized US laboratories (Clinical Biochemical Genetic Laboratories) devoted to the diagnosis and management of IEMs, and the United States has accredited training for clinical biochemical genetic laboratory directors, overseen by the American Board of Medical Genetics and Genomics, that aims to ensure excellence among laboratory directors through continuing education, among other activities. International laboratories may not have access to accredited training programs. Additionally, the distribution of PT specimens may face sporadic delays occurring during shipment to distant destinations thereby affecting sample integrity and impacting interpretation. Indeed, participating laboratories are requested to ensure that specimens arrive frozen and within their storage requirements. Nevertheless, adherence to this request relies on self-reported observations that may be hindered by storage, unpacking, and repackaging at custom office locations. Finally, rare disease incidence differences among populations worldwide may lead to the occurrence that a common genetic condition in one part of the world is seldom observed, if at all, in another region. Hence, laboratories can have divergent experiences with the full spectrum of IEMs. Thus, difference in performance may be due to a combination of these or other factors.

Because multiple factors probably contributed to performance variance for both US and international laboratories, a central function of PT is to prompt assay troubleshooting, process refinement, and quality improvement when failures do occur. This includes prompting laboratories to review their processes for analyte extraction and derivatization, method calibration, and instrument optimization. Analytic errors may also reflect limitations in available laboratory methods, such as for MPS screening and fractionation where assay shortcomings are well recognized^18^ and addressed by emerging new LC-MS/MS methods.^19, 20^ Some errors may result from constraints inherent to the PT process itself, where lower estimates of clinical sensitivity may be due to the lack of supporting clinical information or results of other laboratory tests. This underscores the importance of clinical history when interpreting analytic findings, as pointed out by other groups.^21^ Finally, because laboratories submit PT results outside of their normal data transmission and reporting protocols, inadvertent clerical errors may also occur.

Incorrect interpretations also may have resulted from the strict requirement to report a specific diagnosis, even in cases for which additional testing may be warranted in actual practice. For example, a suspected case of MCAD deficiency identified by OA analysis typically requires additional confirmation by AC analysis, and positive MPS screening and fractionation results requires confirmation by enzyme and/or DNA testing.^21^ Responses representing an incorrect diagnosis (as opposed to a “normal” response in the face of a true abnormality) were classified as false-negative results for this report. Although considered a PT failure, in practice an incorrect diagnosis still is likely to be followed by repeat testing, additional studies, or gathering of additional clinical or family history. Indeed, some diagnoses included in PT schemes, such as carnitine uptake defect (an AC challenge) or glycine encephalopathy (a plasma AA challenge), may have been particularly challenging since these conditions are more appropriately diagnosed in conjunction with other tests (free and total carnitine and cerebrospinal fluid AA analysis, respectively). Nonetheless, despite the inherent constraints of PT, a correct diagnosis could most often unequivocally be assigned based on results of a single test.

Among other PT programs available for BGLs, the European consortium ERNDIM provides challenges for qualitative OA and urine MPS schemes that are similar to the CAP/ACMG program. As a comparison, reported ERNDIM performance (see www.erndim.org/home) for 2015 showed that accuracy ranges from 60 to 89% for MPS and 85 to 100% for OA. Hence, the PT performance of laboratories observed in the CAP/ACMG scheme is similar to those found in other programs.

From this study, current clinical biochemical genetic laboratories demonstrated good overall performance on PT. Remaining challenges for laboratories include the need for standardizing laboratory methods and reagents, and ensuring that ongoing education enhances the awareness of genetic disorders, and their diagnostic profiles, thereby benefiting patient care.

## Figures and Tables

**Table 1:** Estimates of analytic sensitivity for the four schemes included in the biochemical genetic laboratories proficiency testing program stratified by laboratory location

**Scheme**	**Location**	**Analytic true positive**^a^	**Analytic false negative**^b^	***P*** **value**^c^
Amino acids	US	95.1% (1,122/1,180) (93.9–96.3%)	4.9% (58/1,180) (3.7–6.1%)	<0.001
	International	89.4% (430/481) (86.7–92.2%)	10.6% (51/481) (7.9–13.3%)	
Organic acids	US	93.5% (942/1,007) (92.0–95.1%)	6.5% (65/1,007) (4.9–8.0%)	0.043
	International	90.4% (369/408) (87.6–93.3%)	9.6% (39/408) (6.7–12.4%)	
Acylcarnitine profile	US	93.1% (471/506) (90.9–95.3%)	6.9% (35/506) (4.7–9.1%)	0.060
	International	88.9% (192/216) (84.7–93.1%)	11.1% (24/216) (6.9–15.3%)	
Mucopolysaccharides	US (Screening)	93.0% (318/342) (90.3–95.7%)	7.0% (24/342) (4.3–9.7%)	0.056
	International (Screening)	88.2% (179/203) (83.7–92.6%)	11.8% (24/203) (7.4–16.3%)	
	US (Fractionation)	91.4% (213/233) (87.8–95.0%)	8.6% (20/233) (5.0–12.2%)	0.0010
	International (Fractionation)	79.0% (94/119) (71.7–86.3%)	21.0% (25/119) (13.7–28.3%)	

a Proportion of correct abnormal analyte identified (analytic sensitivity).

b Proportion of incorrect abnormal analyte identified, or no abnormal analyte identified.

c Comparison of analytic sensitivity between US and international participants.

**Table 2:** Estimates of clinical sensitivity for the four schemes included in the biochemical genetic laboratories proficiency testing program stratified by laboratory location, along with the rate of the two types of false-negative errors

		**True positive**	**False negative**		
**Schemes**	**Location**	**Correct diagnosis**^a^	**Incorrect disorder identified**^b^	**No disorder found: normal**^c^	***P*** **value**^d^
Amino acids	US	92.9% (1,103/1,188) (91.4–94.3%)	4.7% (56/1,188) (3.5–5.9%)	2.4% (29/1,188)^e^ (1.6–3.3%)	<0.001
	International	86.5% (411/475) (83.5–89.6%)	10.3% (49/475) (7.6–13.1%)	3.2% (15/475) (1.6–4.7%)	
Organic acids	US	92.9% (947/1019) (91.4–94.5%)	4.2% (43/1019) (3.0–5.5%)	2.9% (29/1019) (1.8–3.9%)	<0.001
	International	84.7% (354/418) (81.2–88.2%)	8.4% (35/418) (5.7–11.0%)	6.9% (29/418) (4.5–9.4%)	
Acylcarnitine profile	US	90.1% (462/513) (87.5–92.7%)	7.2% (37/513) (5.0–9.5%)	2.7% (14/513) (1.3–4.1%)	0.0082
	International	83.2% (188/226) (78.3–88.1%)	8.8% (20/226) (5.1–12.6%)	8.0% (18/226) (4.4–11.5%)	
Mucopolysaccharides	US	88.3% (227/257) (84.4–92.3%)	8.9% (23/257) (5.5–12.4%)	2.7% (7/257) (0.7–4.7%)	<0.001
	International	72.1% (106/147) (64.69–79.4%)	20.4% (30/147) (13.9–26.9%)	7.5% (11/147) (3.2–11.7%)	

a Proportion of participants correctly identifying the targeted disorder (clinical sensitivity).

b Proportion of participants incorrectly identifying a disorder other than the intended target.

c Proportion of participants incorrectly providing a normal response for a challenge with a specific disorder.

d Comparison of clinical sensitivity between US and international participants.

e All false-negative results occurred prior to 2011.
