# Supplementary material for: CAP/ACMG proficiency testing for biochemical genetics laboratories: a summary of performance
Source: Genet Med. 2017 Jun 29;20(1):83–90. doi: 10.1038/gim.2017.61 (PMC5763156; doi:10.1038/gim.2017.61)
Supplement: Supplemental Materials [file gim201761x1.docx]

**SUPPLEMENTAL MATERIALS**

**Table S1. Individual sample challenges for the Biochemical Genetics Proficiency Testing Survey from 2004-B through 2014-B: Amino Acids (AA), Organic Acids (OA), Acylcarnitine (AC) and Mucopolysaccharidosis (MPS).**

**Figure S1. Colored Heat maps for CAP BGL external proficiency testing for Amino Acids (AA) between 2004-B and 2014-B, stratified by geographic location.**

**Figure S2. Colored Heat maps for CAP BGL external proficiency testing for Organic Acids (OA) between 2004-B and 2014-B, stratified by geographic location.**

**Figure S3. Colored Heat maps for CAP BGL external proficiency testing for Acylcarnitine (AC) between 2004-B and 2014-B, stratified by geographic location.**

**Figure S4. Colored Heat maps for CAP BGL external proficiency testing for Mucopolysaccharidosis (MPS) between 2004-B and 2014-B, stratified by geographic location.**

**Table S1. Individual sample challenges for the Biochemical Genetics Proficiency Testing Survey from 2004-B through 2014-B: Amino Acids (AA), Organic Acids (OA), Acylcarnitine (AC) and Mucopolysaccharidoses (MPS)**

| **Challenge** | **AA** | **OA** | **AC** | **MPS** |
| --- | --- | --- | --- | --- |
|  |  |  |  |  |
| 2004 B | Alpha-aminoadipic acidemia | Ethylmalonic encephalopathy | MCAD deficiency | MPS I / Hurler syndrome |
| 2005 A | Maple-syrup urine disease | Fumarase deficiency | Glutaric acidemia-I | MPS III / Sanfilippo syndrome |
| 2005 B | Lysinuric protein intolerance/hyperlysinuria | Malonic acidemia | Methylmalonic aciduria /PA | Normal |
| 2006 A | Homocystinuria | Normal (not included) | 2-Methylbutyryl-CoA dehydrogenase | MPS VII / Sly syndrome |
| 2006 B | Hyper Beta-Alaninemia (Cohen Syndrome) | Isobutyryl CoA dehydrogenase deficiency | 3-Methylcrotonyl-CoA carboxylase deficiency | Normal |
| 2007 A | Hyperlysinemia | Hyperoxaluria type II | VLCAD deficiency | MPS III / Sanfilippo syndrome |
| 2007 B | Argininosuccinic aciduria | Propionic acidemia | VLCAD deficiency | MPS IV / Morquio syndrome |
| 2008 A | Methylmalonic aciduria / homocystinuria, CblC | Methylmalonic aciduria /Homocysteinuria, CblC | Malonic acidemia | MPS VII / Sly syndrome |
| 2008 B | Primary lactic acidemia | Glutaric academia type II | SCAD deficiency | Normal |
| 2009 A | Hyperornithinemia | 5-Oxoprolinuria / Glutathione synthase deficiency | LCHAD deficiency | MPS II / Hunter syndrome |
| 2009 B | Non-ketotic hyperglycinemia | Ornithine transcarbamylase deficiency | Carnitine uptake defect | MPS III / Sanfilippo syndrome |
| 2010 A | Cystinuria | MCAD deficiency | Beta-Ketothiolase deficiency | MPS II / Hunter syndrome |
| 2010 B | Hyperprolinemia type II | Mevalonic aciduria | Glutaric acidemia type I | MPS IV / Morquio syndrome |
| 2011 A | Hyperornithinemia | Fumarase deficiency | 3-Methylcrotonyl-CoA carboxylase deficiency | MPS VII / Sly syndrome |
| 2011 B | MSUD | Isovaleric aciduria | LCHAD deficiency | Normal |
| 2012 A | Citrullinemia | Glutaric academia type I | Malonic acidemia | MPS III / Sanfilippo syndrome |
| 2012 B | PKU | Methylmalonic aciduria | SCAD deficiency | Normal |
| 2013 A | Normal plasma | MCAD deficiency | Glutaric acidemia type I | MPS II / Hunter syndrome |
| 2013 B | Nonketotic hyperglycinemia | 3-Methylcrotonyl-CoA carboxylase deficiency | Normal plasma | Normal |
| 2014 A | Arginase deficiency | Glutaric acidemia type I | VLCAD deficiency | MPS III / Sanfilippo syndrome |
| 2014 B | Cystinuria | Propionic acidemia | Glutaric acidemia type II | MPS IV / Morquio syndrome |

**Figure S1. Colored Heat maps for CAP BGL external proficiency testing for Amino Acids (AA) between 2004-B and 2014-B, stratified by geographic location**

Paired columns indicate individual challenges (A=analytic results and I-interpretation). Rows indicate individual laboratory responses. The data are sorted from top to bottom by the number of challenges graded. Green indicates a correct response. Red (under interpretation or analyte) indicates an abnormal analyte/clinical finding was reported, but it was not the correct one. A yellow square (under interpretation) indicates that the clinical interpretation was normal (false negative) when the correct response was an abnormality. White indicates no response and these often occur in pairs. The gray columns under the 2013-A distribution indicate the challenge was not suitable for grading (sample degradation prior to shipment). Figure S1a shows the data for the 50 International participants and Figure S1b shows the data for the 109 US participants.

**Figure S1a. Amino Acid Heat map for 50 International Participants**

**Figure S1b. Amino Acid Heat map for 109 US Participants**

**Figure S2. Colored Heat maps for CAP BGL External Proficiency Testing for Organic Acids (OA) between 2004-B and 2014-B, stratified by geographic location**

Paired columns indicate individual challenges (A=analytic results and I=interpretation). Rows indicate individual laboratory responses. The data are sorted from top to bottom by the number of challenges graded. Green indicates a correct response. Red (under interpretation or analyte) indicates an abnormal analyte/clinical finding was reported, but it was not the correct one. A yellow square (under interpretation) indicates that the clinical interpretation was normal (false negative) when the correct response was an abnormality. White indicates no response and these often occur in pairs. Figure S2a shows the data for the 40 International participants and Figure S2b shows the data for the 73 US participants.

**Figure S2a. Organic Acid Heat map for 40 International Participants**
**Figure S2b. Organic Acid Heat map for 73 US Participants**

**Figure S3. Colored Heat maps for CAP BGL external proficiency testing for Acylcarnitine (AC) between 2004-B and 2014-B, stratified by geographic location**

Paired columns indicate individual challenges (A=analytic results and I-interpretation). Rows indicate individual laboratory responses. The data are sorted from top to bottom by the number of challenges graded. Green indicates a correct response. Red (under interpretation or analyte) indicates an abnormal analyte/clinical finding was reported, but it was not the correct one. A yellow square (under interpretation) indicates that the clinical interpretation was normal (false negative) when the correct response was an abnormality. Prior to 2009, the stored data was not sufficient to distinguish the ‘yellow’ from ‘red’ errors. White indicates no response and these often occur in pairs. Figure S3a shows the data for the 23 International participants and Figure S3b shows the data for the 42 US participants.

**Figure S3a. Acylcarnitine Heat map for 23 International Participants**

**Figure S3a. Acylcarnitine Heat map for 42 US Participants**

**Figure S4. Colored Heat maps for CAP BGL external proficiency testing for Mucopolysaccharidosis (MPS) between 2004-B and 2014-B, stratified by geographic location**

Columns are grouped into threes and together indicate an individual challenge (**S**creening, **F**ractionation and **I**nterpretation). Rows indicate individual laboratory responses. The data are sorted from top to bottom by the number of challenges graded. Green indicates a correct response. Red (under interpretation or analyte) indicates an abnormal analyte/clinical finding was reported, but it was not the correct one. A yellow square (under interpretation) indicates that the clinical interpretation was normal (false negative) when the correct response was an abnormality. White indicates no response, and these often occur in pairs. The gray columns indicate the challenge was not suitable for grading (no consensus reached). Blue background in the challenge indicates it is a specificity challenge (normal sample). Figure S4a shows the data for the 30 International participants and Figure S4b shows the data for the 42 US participants.

**Figure S4a. Mucopolysaccharides Heat map for 30 International Participants**

**Figure S4b. Mucopolysaccharides Heat map for 42 US Participants**
